# The Voluntary System of Medical Education

**Published:** 1854-11

**Authors:** 


*The Voluntary System of Medical Education* Instituted by the Independent Medical School of Pensylvania, Chartered May 8th, 1854: King & Baird, Printers, Philadelphia.

The above is the title of a pamphlet of 20 pages, containing some just criticisms on the present system of Medical Education, and explaining the nature and objects of what is styled the "Independent Medical School of Pennsylvania."

The proposed organization is not to be a school for giving instruction or lectures, but solely for the purpose of securing an independent board of examiners before whom the candidate for Medical honors can present himself and receive a thorough examination without reference to the place where he may have pursued his studies or attended on lectures. We infer from the pamphlet that a charter has been obtained granting all the powers necessary for carrying into effect such an institution.

An attempt of a similar character was made in Philadelphia, so early as the year 1837; and was then advocated by a very large proportion of the profession in that city. But the legislature then refused to grant them the necessary charter. (See Eclectic Journal, Med. vol. 1. p. 80.) We hope the present effort will meet with better success; for we hail every movement that tends to sever the present connection between the business of *teaching* medicine and granting diplomas which serve as licenses to practice.

We have long contended that the several medical schools of our country could never be thrown fully upon their actual merits, as institutions of learning and sound instruction, so long as the privilege of conferring degrees placed the most insignificant school on the same level, in this respect, with the most renowned. But the establishment of ever so many *Independent Boards of Examination* will not remedy some of the most prominent objections to the present system of medical education. Take, for instance, the objection stated very clearly in the following paragraph from the pamphlet before us:

"Out of the customary limitation to a *duplicated session* of four or five months, (the two sessions that make up the term of public instruction, are in fact, but copies and representations of each other,) *there grows an unavoidable confusion of study*—a mixture without order, relation, or natural sequence and dependency. Anatomy, for instance, which certainly ought to have some fixed place in the programme of tuition, corresponding to its natural order in study, is taught in a tangled coil of surgery, physiology, therapeutics, and obstetrics! The surgeon begins, perhaps, with inflammation, and the chair of practice opens on fevers, while the anatomist is still lecturing on the *bones*. And amputations, the action of remedies upon the vascular and nervous systems, and the mechanism of parturition; are under discussion in their several chairs, before the anatomist has reached a muscle, artery or nerve, involved in the prelections of his fellow-professors!"

This crowding of the whole field of Medical Science upon the mind of the student without method or natural order, during a limited session of college instruction, has long been felt as a radical fault. But the remedy proposed both in the pamphlet and elsewhere, which consists in *simply lengthening the college terms*, is wholly futile and inadequate. Here was the great error of the American Medical Association. Its members saw clearly the absurdity of crowding the field of medical study into the brief period of four months, and hence one of their first acts was to recommend the addition of *two months* to the college term. But in doing this they left the same confusion, the same daily crowding of diverse branches upon the mind, regardless of their natural sequence, as before. Consequently it only subjected the student to a more protracted endurance of the same difficulties as in the short sessions; and the recommendation proved an entire failure. If the Association had, with the same unanimity, recommended the annual college session to be extended to *nine* months, instead of six; and then subdivided it into *terms* of three months each, with a division of the science of medicine into *twelve* departments—four of which should be taught in each of the *terms* of the college session; they might have so grouped the several departments or branches that all students during the first year of study should attend one term of *four* branches, presented in four lectures a day for three months, thereby giving ample time for practical dissections and study—those in the second year of study should attend another term of *four* branches, three months, during which they should still dissect and also attend hospital for the special purpose of studying Pathology—and those in the third and last year of pupilage, having now laid a good foundation should attend the *third* term of the college session, which should embrace the great remedial or directly practical branches with clinical instruction both for its Pathology and Therapeutics.

This would place the several departments of medical study in something like their natural order of sequence and dependence, and wonld secure a most desirable degree of method and thoroughness in our system of medical education, without any increased tax on the student's time or pecuniary resources. If such a system of college instruction was adopted together with the establishment of independent boards of censors for confering degrees, it would soon place every college entirely on its merits and resources for instruction, and give to college competition a widely different tendency, in some respects, from what it has possessed during the last half century. In the meantime we would commend to the attention of the friends of the "Voluntary System," in Pennsylvania, the concluding chapter of a little (and much abused work) we published a few years since "On Medical Education." D.

